# Face familiarity promotes stable identity recognition: exploring face perception using serial dependence

**DOI:** 10.1098/rsos.160685

**Published:** 2017-03-01

**Authors:** Rebecca Kok, Jessica Taubert, Erik Van der Burg, Gillian Rhodes, David Alais

**Affiliations:** 1 The School of Psychology, The University of Sydney, Camperdown, New South Wales 2006, Australia; 2 Department of Cognitive Psychology, Vrije Universiteit Amsterdam, Amsterdam, The Netherlands; 3 The School of Psychology, The University of Western Australia, Crawley, Western Australia 6009, Australia

**Keywords:** face perception, object continuity, face recognition, inter-trial effects

## Abstract

Studies suggest that familiar faces are processed in a manner distinct from unfamiliar faces and that familiarity with a face confers an advantage in identity recognition. Our visual system seems to capitalize on experience to build stable face representations that are impervious to variation in retinal input that may occur due to changes in lighting, viewpoint, viewing distance, eye movements, etc. Emerging evidence also suggests that our visual system maintains a continuous perception of a face's identity from one moment to the next despite the retinal input variations through serial dependence. This study investigates whether interactions occur between face familiarity and serial dependence. In two experiments, participants used a continuous scale to rate attractiveness of unfamiliar and familiar faces (either experimentally learned or famous) presented in rapid sequences. Both experiments revealed robust inter-trial effects in which attractiveness ratings for a given face depended on the preceding face's attractiveness. This inter-trial attractiveness effect was most pronounced for unfamiliar faces. Indeed, when participants were familiar with a given face, attractiveness ratings showed significantly less serial dependence. These results represent the first evidence that familiar faces can resist the temporal integration seen in sequential dependencies and highlight the importance of familiarity to visual cognition.

## 1. Introduction

Face perception plays an integral role in the way we communicate with others. However, our ability to accurately recognize faces across changes in context is limited; we are surprisingly bad at recognizing and matching unfamiliar faces [1–3]. Participants are much slower and less accurate when attempting to match personally unfamiliar faces than familiar faces [4–8]. Unfamiliar face matching remains poor even when high-quality images are used [9,10]. By contrast, overwhelming evidence indicates that participant recognition and matching performance with familiar faces survives a remarkable number of image transformations that reliably disrupt performance with unfamiliar faces, such as changes in background [11,12], viewpoint [8,9,13–19] and expression [14]. Familiar faces are more quickly and accurately recognized than unfamiliar faces even in challenging viewing conditions [20–22]. The striking performance differences between familiar and unfamiliar faces are particularly remarkable given they belong to the same class of visual objects (i.e. faces) and share the same configuration of features and shows that the underlying system is able to capitalize on past experience to promote stable identity recognition.

Recent studies of the perceptual system's maintenance of visual continuity provide insight into the stability conferred by familiar face representations. Evidence indicates that our ability to perceive stable representations of objects despite frequent fluctuations in visual input (e.g. by occlusion, blinks or saccades) is supported by a mechanism that integrates information across time to stabilize perception, known as serial dependence. Studies on visual orientation [23], numerosity [24], auditory frequency [25] and multisensory timing [26–28] have all shown that the percept of an object or event depends on the immediately preceding stimulus. Typically, this serial dependence is assimilative and follows brief stimulus presentations, while extended stimulus exposures (as little as 5 s) result in classical repulsive after-effects whereby subsequent stimuli are repelled away from previous stimuli [23]. Repulsive after-effects are well documented for low-level features such as orientation [29] and for high-level stimuli such as faces [30–32].

Serial dependence can also occur with high-level stimuli such as faces. Liberman *et al*. [33] recently studied face identity using briefly presented faces (500 ms) drawn from morphed continua and found that perceived identity on a given trial was systematically biased towards the identity seen in the preceding trial. This inter-trial effect was evident across rotations in viewpoint and even after controlling for a potential response bias by having participants not respond on a randomly selected 50% of the trials, thereby removing the previous response itself as a contributing factor.

In addition to face identity, attractiveness is a key aspect of face perception with well-documented significance in social interactions, particularly in judgements of a person's health, romantic viability and intelligence, even when told not to consider it or when more objective evidence should drive our judgements (for reviews, see [34,35]). Facial attractiveness has also been studied in serial dependency experiments and consistent with findings for face identity, perceptual assimilation effects have been observed in sequences of face attractiveness judgements [36–38]. Using discrete Likert-scale ratings, a face was rated more attractive when participants rated the previous face as attractive compared with when they rated it as unattractive. Recently, a similar serial dependence finding was reported for binary attractiveness judgements [39].

Pegors *et al*. [40] investigated whether this assimilative face perception effect is driven by a response bias or relies on a low-level perceptual effect caused by the attractiveness of the preceding face. Participants viewed a face and rated in an alternating order either its attractiveness or the darkness of that face's hair. Attractiveness ratings were compared with the previous response, regardless of the task, and to the previous ‘stimulus value’ (an attractiveness rating made by independent raters). Pegors *et al*. found that participant responses assimilated from trial to trial, despite the alternating task requirements, but that attractiveness on any given trial was driven away from the average attractiveness of the previous face (determined by an independent sample of participants), thereby ostensibly demonstrating that the inter-trial effect resulted from response bias rather than a perceptual phenomenon related to the previous stimulus's attractiveness.

Several factors argue against a response-bias interpretation of Pegors *et al*.'s data [40]. First, participants performed at chance levels when asked to recognize faces seen in hair-darkness trials, and performed significantly better with faces seen in attractiveness trials, indicating that relatively little attention was paid to identity in hair-darkness trials. Indeed, encouraging participants to attend to hair could explain why identity was not processed. External features such as hair are commonly thought to distract one from successful face recognition, especially given the inner face advantage in familiar faces [8,41]. Thus if serial dependence were indeed the underlying mechanism stabilizing identity perception, there would be no opportunity for it to arise here. Also, the length of stimulus presentation (1–4 s) was considerably longer than exposures used in previous studies of inter-trial effects (rarely reaching 1 s) and this may have produced classical adaptation after-effects which have been shown to override serial dependence when stimulus presentation is extended from 500 ms to 5 s [23].

The present research tests whether face familiarity will diminish the impact of serial dependence because representations of familiar faces are less vulnerable to superficial changes in the visual signal (for a review, see [42]). We asked participants to rate familiar and unfamiliar faces using a sliding bar to represent a continuous attractiveness scale. The position and size of face stimuli were jittered from trial to trial to minimize local adaptation. In one experiment, we investigated familiarity by training participants on a set of faces, thus controlling the amount of experience with each stimulus [43,44]. In a second experiment, we ran the same task with famous faces, trading off experimental control for ecological validity, predicting a significantly reduced inter-trial effect for famous faces. In both experiments, we predict a positive serial dependency (i.e. assimilation): faces should appear more attractive after an attractive face, and less so after an unattractive face. For familiar faces, this inter-trial effect should be less susceptible to the preceding face's attractiveness.

## 2. Experiment 1

To test whether visual familiarity with faces affects inter-trial attractiveness effects, participants were asked to learn a set of faces (the ‘familiar’ faces) and were tested for recognition of these faces, before rating the attractiveness of a mix of familiar and novel faces presented in a random order. We expected that faces would be rated as more attractive after attractive faces, and less attractive after unattractive faces [45], but that this effect would be significantly reduced for familiar faces for which the participants had the opportunity to build a robust representation [42].

## 3. Experiment 1. Method

### 3.1. Participants

Twenty-two participants were recruited for the experiment and were paid AU $20/h. One participant felt they were unable to complete the experiment, leaving 21 participants (13 female) who completed all tasks required. The target sample size was chosen based on independent research that investigated similar effects using the same paradigm [45]. Each participant gave their written consent prior to completing the experiment. All participants reported normal or corrected-to-normal vision and were naive as to the purpose of the experiment.

### 3.2. Experimental stimuli

Stimuli consisted of the first 220 colour photographs in a set organized by filename order of frontward faces with neutral expressions measuring 320 × 420 pixels used by Rhodes *et al*. [46]. All faces were photographed from a fixed distance of 190 cm under uniform lighting and were initially novel to participants.

From the 220 faces in the main image set, 40 faces were randomly chosen to create two different sets of 20 ‘familiar’ faces (Set A and Set B). Eleven participants learned Set A, while 10 learned Set B. For every participant, the remaining 200 faces in the main image set were then divided into two sets of ‘novel’ face stimuli—a set of 60 novel faces were used only in the recognition task, while the remaining 140 novel faces were used only in the adaptation task. An equal number of male and female faces were used in each set of face stimuli to avoid any gender bias as past research has found a tendency of participants to see same-gender faces as more attractive [47].

Participants completed the experiment in a dimly lit curtained booth. The experiment was programmed in Matlab version R2010a using the Psychophysics Toolbox 3 [48,49]. The program was run on an Apple Mac Pro running Mac OSX Lion v. 10.7.5. Participants were seated approximately 57 cm in front of a CRT monitor (18 inch viewable screen size) set at a screen resolution of 1024 × 768 pixels with a refresh rate of 100 Hz at which distance the faces subtended approximately 7 × 10 degrees of visual angle. Participant input was recorded using an Apple wired USB keyboard.

### 3.3. Experimental design

The experiment employed a 2 × 2 within-subjects design with attractiveness ratings as the dependent measure, meaning that each participant was exposed to four possible conditions while rating faces in the adaptation test stage. The independent variables were the familiarity of a face on a given trial *n* (*current face familiarity*: familiar or novel) and the attractiveness of the face on trial *n* − 1 (attractive or unattractive). Faces were categorized as attractive or unattractive for each participant with reference to their own median attractiveness rating—faces with ratings above their median score were categorized as attractive, while those with scores lower than their median were categorized as unattractive. Since familiar faces were rated multiple times, an attractiveness rating averaged across all instances in the adaptation stage was used for the purposes of their categorization as attractive or unattractive.

### 3.4. Experimental procedure

Participants undertook three tasks during the course of the experiment in a fixed order: (i) learning, (ii) recognition, and (iii) adaptation test stage.

#### 3.4.1. Learning task

The learning stage involved six blocks of 200 trials each (1200 trials in total). Each block took approximately 10 min to complete, and participants were given the opportunity to take a short rest between each one. During this stage, participants completed a sequential two-alternative forced-choice task and were instructed that a recognition task would follow.

**Figure 1.**
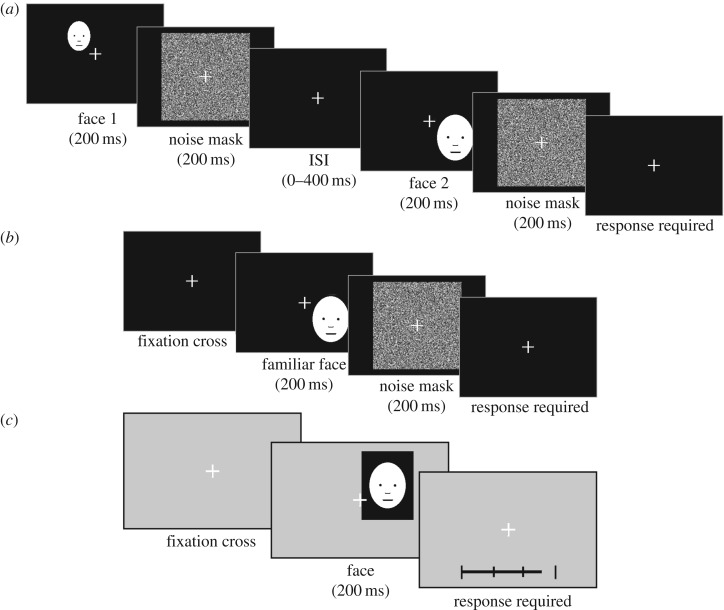
Experimental procedure. (*a*) Sequencing of events for each trial in the learning task after trial initiation. Participants were presented with a random face from their allocated set of familiar faces (Set A or B), a noise mask, a second familiar face and a final noise mask before being required to respond ‘same’ (up-arrow key) or ‘different’ (down-arrow key). A randomly varying ISI of 0–400 ms occurred between presentations of the first noise mask and the second familiar face. (*b*) Sequencing of events for each trial in the recognition task. Once participants initiated a trial, they were presented with a random face from either their allocated set of familiar faces (Set A or B) or a novel face. After a noise mask, participants were required to respond whether the face was learnt (up-arrow key) or not learnt (down-arrow key). (*c*) Sequencing of events for each trial in the adaptation task, ending with a slider for the participants to indicate their attractiveness rating using the left- and right-arrow keys on the keyboard.

Each block began with the presentation of a white fixation cross (1.5° of visual angle in width and height) drawn in the centre of a black background until participants initiated the first trial with a button press. As seen in figure 1*a*, participants were then presented with a face randomly selected from a set of 20 familiar faces (Set A or Set B), followed by a randomly generated noise mask. After a random inter-stimulus interval (ISI) of 0–400 ms, a second familiar face and noise mask were shown. The faces and noise masks were only presented for 200 ms each. After each pair of faces, participants indicated whether the two faces presented were the same or different by using the up- or down-arrow key, respectively. To disrupt any local image adaptation and the use of low-level features in any cognitive matching strategies, stimuli were jittered in size and position by 12.5% of the original image size. The jittering was random except for the constraint that faces in consecutive trials were never presented at the same size. After participants made a response, the next trial was initiated after a random inter-trial interval (400–800 ms).

#### 3.4.2. Recognition task

Each trial began with the presentation of a face for 200 ms, followed by a mask for another 200 ms (figure 1*b*). Subsequently, participants were asked to determine whether the face was familiar (i.e. seen during the learning phase) or not by pressing the up- or down-arrow key, respectively. After a response was made, the next trial was initiated after a random inter-trial interval (400–800 ms). In total, 80 different faces were shown, comprising the 20 familiar faces (shown during the learning phase) and 60 unfamiliar faces (never seen before). In total, each face was repeated five times, making a total of 400 trials during the recognition phase. Face stimuli were again jittered in size and location, so that they never appeared in the same size, or location, on consecutive trials.

#### 3.4.3. Adaptation task

In the final phase of the experiment, participants completed seven blocks of an adaptation task in which they rated the attractiveness of each given face. Each block presented the 20 ‘learned’ faces and 20 ‘novel’ faces (in each of the seven blocks, a different set of 20 faces were drawn from the 140 ‘previously unseen’ faces remaining in the main stimulus set) in a random sequence for 200 ms each (figure 1*c*). In any extra time remaining after the seven blocks, participants continued to repeat blocks for the purpose of recording more ratings for familiar faces—this would allow derivation of a more stable average attractiveness score for each familiar face. Otherwise, the participants' ratings of face attractiveness on these trials were not used for the purpose of serial dependence analyses.

On each trial, immediately after a face stimulus disappeared, participants were presented with a grey screen and a black horizontal sliding scale measuring 200 pixels across at the bottom of the screen (from left to right, the scale ranged from very unattractive to very attractive), on which they indicated their attractiveness rating using the left- and right-arrow keys (figure 1*c*). The scale had a vertical marker between two endpoints corresponding to minimum and maximum ratings and at each quarter. Participants pressed the space bar when they were satisfied with their rating. Subsequently, the next trial was initiated after a random inter-trial interval (400–800 ms). Furthermore, the slide bar was set at a random level at the start of each trial to discourage repetitive settings. Participants were encouraged to rate the faces quickly and to use the entire length of the slide bar as much as possible.

## 4. Experiment 1. Results and discussion

### 4.1. Data preparation

For each participant, mean attractiveness ratings were calculated for each of the eight possible conditions described in §3.3. First, adaptation trials were binned into two groups according to the familiarity of the current trial: familiar or unfamiliar. Responses to the first trial of each block were necessarily excluded because they did not have a preceding trial.

**Figure 2.**
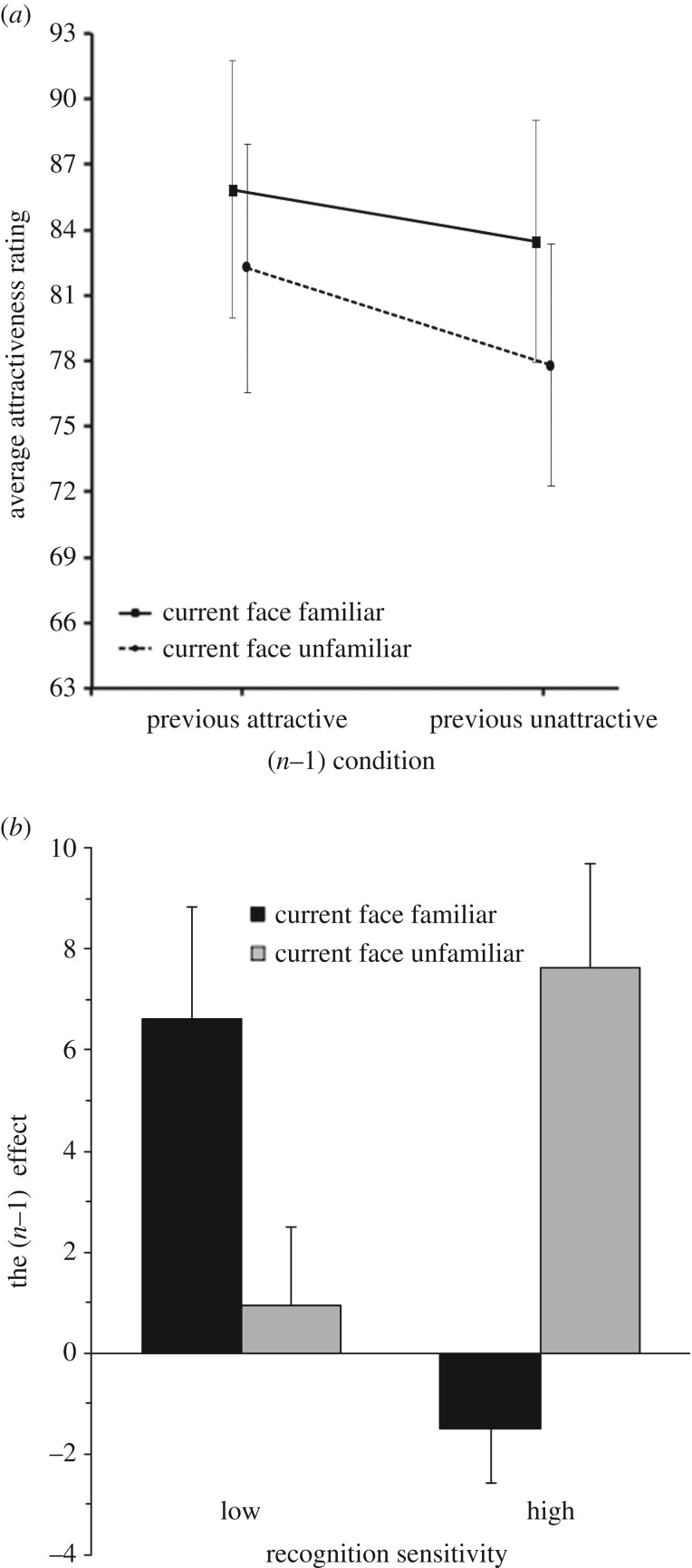
Results of Experiment 1. (*a*) Average attractiveness ratings for familiar and unfamiliar faces as a function of attractiveness of the previous face in Experiment 1, with minimum and maximum ratings of 0 and 200, respectively. Error bars represent the standard error of the mean. (*b*) The mean inter-trial attractiveness effect for familiar and unfamiliar faces, for the low-sensitivity (*n* = 10) and high-sensitivity (*n* = 11) recognition groups in Experiment 1. Error bars represent the standard error of the mean.

The trials were then further binned according to whether the face in the preceding trial was ‘attractive’ or ‘unattractive’ to determine whether attractive faces were more likely to follow attractive faces than unattractive faces, and *vice versa*. The scores in each of the resulting four bins were averaged to derive the mean attractiveness ratings for each of the four possible conditions. Figure 2*a* plots the overall mean of these ratings across all 21 participants.

### 4.2. Data analyses

#### 4.2.1. Recognition task

The average accuracy in the recognition task was 90.3% (s.d. = 0.11, average reaction time = 590 ms). To confirm that the participants were performing above chance (50%), we used a one-sample *t*-test, *t*_20_ = 16.4, *p* < 0.001, *d* = 7.33, two-tailed. The average hit rate and false alarm rates were 87.12% (s.d. = 12.29) and 6.5% (s.d. = 11.91), respectively, corresponding to a *d*-prime value of *d*′ = 2.65.

#### 4.2.2. Serial dependence analyses

Figure 2*a* plots the mean attractiveness rating as a function of attractiveness of the previous face for both familiar and unfamiliar current faces. The mean attractiveness rating on the 200-pixel attractiveness scale across all trials of all participants was 81.9 (s.d. = 45.4). A 2 × 2 repeated-measures ANOVA was conducted on the mean attractiveness with current face familiarity (familiar versus unfamiliar) and attractiveness of the *n* − 1 face (attractive versus unattractive) as within-subject variables.

The analysis yielded a significant attractiveness *n *− 1 effect, *F*_1, 20_ = 7.78, *p* = 0.011, $\eta_{p}^{2}=0.280$, such that faces were perceived as being more attractive when the preceding face was attractive (*M* = 84.1, s.d. = 26.2) than when the preceding face was unattractive (*M* = 80.7, s.d. = 24.8). This observation is consistent with previous studies showing that the attractiveness of a face depends on the attractiveness of the recently observed face [39]. There was also a main effect of current face familiarity, *F*_1, 20_ = 6.84, *p* = 0.017, $\eta_{p}^{2}=0.255$, indicating that familiar faces (*M* = 84.7, s.d. = 25.9) were rated as more attractive than novel faces (*M* = 80.0, s.d. = 25.6). By contrast, the interaction between the inter-trial attractiveness effect and current face familiarity did not reach significance, *F*_1, 20_ = 0.76, *p* = 0.392, $\eta_{p}^{2}=0.037$, suggesting that serial dependence is not affected by face familiarity.

However, the participants' ability to learn faces differed substantially across the individuals, as indicated by the range of *d*-prime scores (min = 0.1, max = 2.6). We therefore decided to conduct a *post hoc* analysis taking into account subject performance in the recognition task, using *d*′ as a convenient performance measure. The *d*′ scores obtained from the recognition task were taken to reflect each participant's familiarity with the stimulus set. To simplify analyses, the four possible conditions were reduced to two based on current face familiarity: familiar and novel. This was done by collapsing the two levels of the *n *− 1 effect (attractive or unattractive) into a single difference score which reflected the magnitude of the inter-trial attractiveness effect itself. Participants were then divided into two groups based on a median split of their sensitivity (*d*′) scores—the lower sensitivity group comprised 10 participants (*M* = 1.58, s.d. = 0.73) and the higher-sensitivity group comprised 11 participants (*M* = 2.96, s.d. = 0.35). The mean inter-trial attractiveness effects for each group can be seen in figure 2*b*.

A 2 × 2 mixed-model ANOVA was then conducted on the mean inter-trial attractiveness effects across all participants with current face familiarity (familiar or novel) as a within-subject variable and participant recognition sensitivity (lower or higher) as a between-subject variable. The analysis yielded no significant difference between the two groups (lower: *M* = 3.8, s.d. = 6.6; higher: *M* = 3.1, s.d. = 8.8; *F*_1, 20_ = 0.08, *p* = 0.782, $\eta_{p}^{2}=0.004$). Nor was there a main effect of current face familiarity (familiar: *M* = 2.8, s.d. = 8.7; novel: *M* = 4.4, s.d. = 6.8; *F*_1, 20_ = 0.97, *p* = 0.337, $\eta_{p}^{2}=0.049$). However, there was a significant two-way interaction between current face familiarity and sensitivity, *F*_1, 20_ = 17.95, *p* < 0.001, $\eta_{p}^{2}=0.486$. The source of this interaction effect was determined with a set of paired *t*-tests (two-tailed; *p*-values were Bonferroni corrected). For the group who performed well in the recognition task (i.e. those with a high-sensitivity score), the inter-trial attractiveness effect was significantly smaller for familiar faces (*M* = −1.5, s.d. = 8.5) compared with novel faces (*M* = 7.6, s.d. = 6.9), *MD* = 9.1, *t*_10_ = 3.45, *p* = 0.012, *d* = 1.05. By contrast, for the group who performed poorly in the recognition task (i.e. those with a low-sensitivity score), the *t*-test yielded no significant difference between the familiar (*M* = 6.6, s.d. = 7.1) and novel faces (*M* = 0.9, s.d. = 4.9), *MD* = 5.7, *t*_9_ = 2.55, *p* = 0.062, *d* = 0.83. The absence of a protective effect in the low-sensitivity group suggested that those participants had not sufficiently learned the ‘familiar’ faces. The pattern of results for the low-sensitivity group appeared to be trending in the opposite direction from that expected, with more serial dependence for familiar faces than for novel faces. However, this apparent difference was not borne out by the analysis. Participants in this group may have had a poor understanding of instructions or a lack of motivation, and as such their responses were more unreliable.

Overall, familiar faces appeared to be more immune to the inter-trial effect when participants were better able to distinguish between familiar and novel faces. Thus in Experiment 2, we used the faces of familiar celebrities to ensure a higher level of familiarity with the faces used.

## 5. Experiment 2. Celebrity faces

### 5.1. Experiment 2. Method

#### 5.1.1. Participants

Twenty-one participants from the University of Sydney (15 female) were recruited and completed the experiment, none of whom had participated in the previous experiment. Each participant was paid AU $20/h. All participants had normal or corrected-to-normal vision.

#### 5.1.2. Experimental stimuli

Stimuli were derived from 300 full-colour photographs of faces viewed from the front. All faces were extracted from their original backgrounds and superimposed on a solid black background, as shown in figure 1*a*. Each of the face stimuli measured 320 × 420 pixels and were prepared using the GIMP v2 software.

The ‘familiar’ face stimuli comprised two photos of each of 20 celebrities who were selected from a range of prominent figures, including actors, musicians, politicians, television personalities and comedians. One photo of each celebrity was used in the recognition task, and the remaining photos were used in the adaptation stage. This was done to decrease the likelihood that visual image-matching was driving participants' familiarity with the faces rather than ‘true’ familiarity with the celebrity's identity. Equal numbers of male and female faces were used.

The ‘novel’ face stimuli were derived from photos of prominent figures known in countries outside of Australia where the experiment was completed, including the Netherlands, Turkey, Spain, Greece, France, Italy, Iceland, Poland, Germany, Portugal, Russia, Sweden, Austria and Norway. This was done with the intention that the novel faces would match the familiar faces in attractiveness and distinctiveness (as is often associated with celebrity faces) but would still be unfamiliar to participants in the present experiment.

#### 5.1.3. Experimental procedure

The experiment employed a two-way within-subjects design, again looking at current face familiarity and attractiveness of the previous face. The dependent variable was attractiveness ratings. Experiment 2 followed closely the procedure used in Experiment 1 with the exception of the learning task and the face stimuli used. The present experiment comprised only two tasks: (i) recognition and (ii) adaptation. The recognition task comprised the same task described in Experiment 1 (as shown in figure 1*b*) but instead used the relevant familiar and novel celebrity face stimuli described above. Participants then completed 10 blocks of the adaptation task described in Experiment 1 (as shown in figure 1*c*), in which they rated the attractiveness of each face shown in sequence. Again, familiar and novel faces were replaced with the relevant celebrity face stimuli. If participants finished the required 10 blocks before the booked time for the experiment was finished, participants were also allowed to repeat the same blocks until their experimental session was complete. This meant that we obtained a higher number of attractiveness ratings for each of the familiar faces with which we could draw a more reliable average rating of the familiar faces; ratings for ‘novel’ faces were discarded because they were no longer truly novel once blocks were repeated. The attractiveness ratings from these trials were then discarded after calculating the mean attractiveness ratings of familiar faces, as in Experiment 1, and not included in further analyses of serial dependence.

### 5.2. Experiment 2. Results and discussion

#### 5.2.1. Data preparation

**Figure 3.**
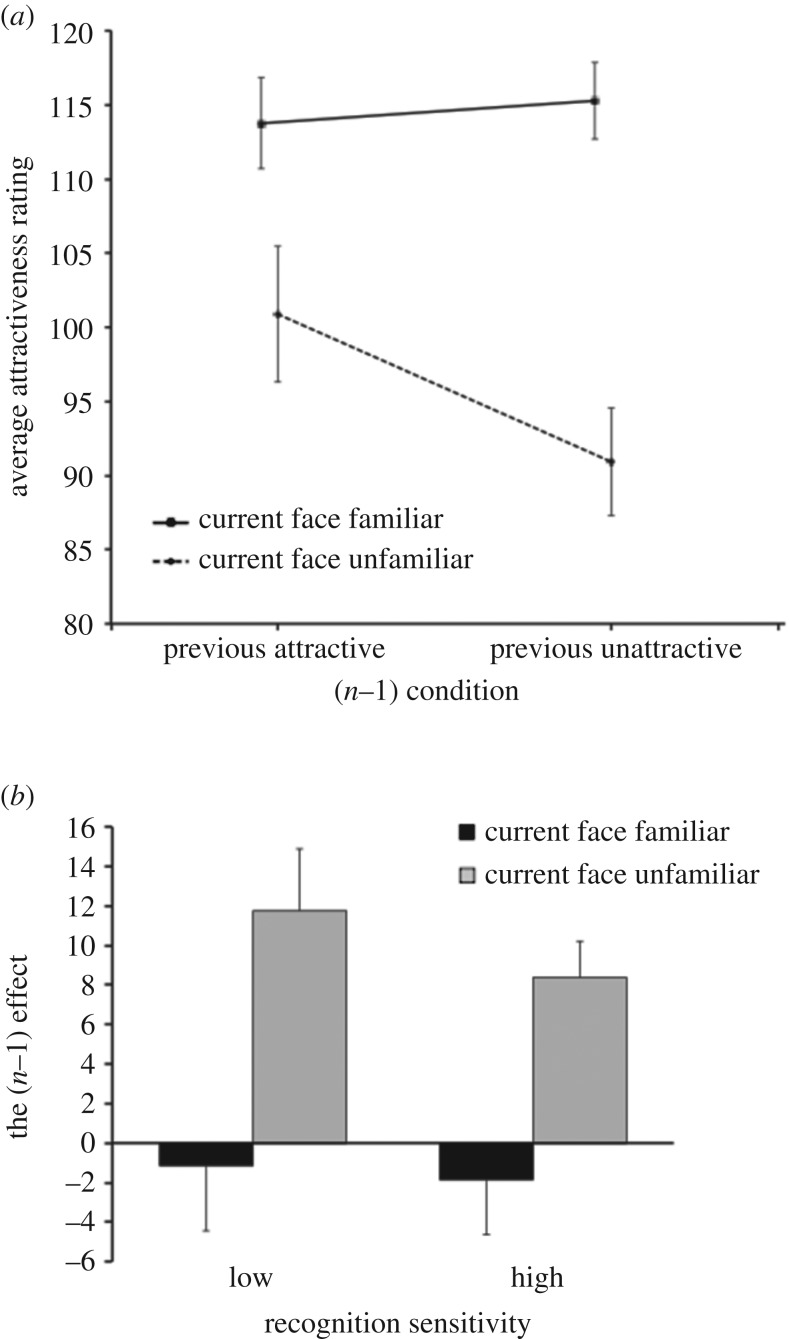
Results of Experiment 2. (*a*) Mean attractiveness ratings for familiar and unfamiliar faces as a function of attractiveness of the previous face in Experiment 2, with minimum and maximum ratings of 0 and 200, respectively. Error bars represent the standard error of the mean. (*b*) The mean inter-trial attractiveness for familiar and unfamiliar faces, for the low-sensitivity (*n* = 10) and high-sensitivity (*n* = 11) groups in Experiment 2. Error bars represent the standard error of the mean.

For the adaptation data, participants' mean attractiveness ratings for each of the four conditions were calculated following the same procedure used in Experiment 1, and are shown in figure 3*a*.

#### 5.2.2. Serial dependence

In the recognition task, the participants were very accurate (90.7%, s.d. = 6.2; performance was above chance level, as confirmed by a one-sample *t*-test, *t*_20_ = 30.08, *p* < 0.001, *d* = 13.45). The overall mean hit rate and false alarm rates were 91.26% (s.d. = 8.2) and 9.83% (s.d. = 8.2), respectively, corresponding to a *d*-prime value of *d*′ = 2.65.

As in Experiment 1, a 2 × 2 repeated-measures ANOVA was initially conducted on all participants' mean attractiveness ratings (shown in figure 3*a*) to determine whether there was a significant effect of the current face (familiar versus novel) and the *n *− 1 condition (attractive versus unattractive). The overall mean attractiveness rating was 104.9, s.d. = 56.0. A significant effect of previous face attractiveness would be evidence of serial dependence occurring, and a significant interaction between current face familiarity and previous face attractiveness would indicate that serial dependence was modulated by familiarity with the target face.

As in Experiment 1, there was a significant main effect of current face familiarity, indicating that familiar faces (*M* = 114.5, s.d. = 12.8) were rated as significantly more attractive than novel faces (*M* = 95.9, s.d. = 19.4), *F*_1, 20_ = 32.95, *p* < 0.001, $\eta_{p}^{2}=0.622$. The analysis also yielded evidence of serial dependence across all participants, in that the expected *n* − 1 effect was found to be significant: previous attractive (*M* = 107.3, s.d. = 18.8); previous unattractive (*M* = 103.0, s.d. = 18.8); *F*_1, 20_ = 7.45, *p* = 0.013, $\eta_{p}^{2}=0.271$. Importantly, these main effects were qualified by a significant interaction between current familiarity and previous attractiveness, *F*_1, 20_ = 23.97, *p* < 0.001, $\eta_{p}^{2}=0.545$, suggesting that familiarity with a face influenced the effect of the *n *− 1 trial. A planned pairwise comparison, with Bonferroni correction, confirmed that ratings of novel faces showed significant serial dependence, *MD* = 10.0, *t*_20_ = 5.62, *p* < 0.001, *d* = 2.02. By contrast, ratings of familiar faces showed no inter-trial attractiveness effect, in line with the hypothesis that familiar faces would be immune to serial dependence, *MD* = 1.5, *t*_20_ = 0.72, *p* = 0.964, *d* = 0.38. Thus no evidence was found to suggest that perception of a familiar face was influenced at all by the previous face.

As in Experiment 1, analyses were rerun taking into account participant recognition sensitivity, and the four mean attractiveness ratings for each participant were collapsed into two *n* − 1 effect measures based on previous and current face familiarity: familiar and novel. Participants were again grouped into ‘lower’ and ‘higher’ sensitivity groups by performing a median split based on their *d*′ scores to allow further determination of whether differences in participants' recognition abilities modulated the influence of familiarity on the *n* − 1 effect. The lower sensitivity group consisted of 10 participants with an average *d*′ score of 1.67 (s.d. = 0.26) and the higher-sensitivity group of 11 participants with an average *d*′ score of 2.54 (s.d. = 0.31). Each group's mean *n* − 1 effects are shown in figure 3*b*.

A 2 × 2 mixed-model ANOVA was conducted as described in Experiment 1 on the size of the *n *− 1 attractiveness effect (figure 3*b*), with current face (familiar or novel) and participant recognition sensitivity (lower or higher) as factors. Only current familiarity had a significant effect, *F*_1, 20_ = 23.33, *p* < 0.001, $\eta_{p}^{2}=0.551$, with significantly smaller *n *− 1 effect for familiar faces (*M* = 1.5, s.d. = 9.6) than for novel faces (*M* = 10.0, s.d. = 8.1) across all participants, regardless of recognition sensitivity. All other *F*s < 0.415, *p*s > 0.52.

## 6. General discussion

The present research yielded two important findings. In both experiments, we found evidence of serial dependence in perceived attractiveness—faces were rated as more attractive when they appeared after attractive faces rather than after unattractive faces. Moreover, this difference in perceived attractiveness was significantly smaller when the current face being rated was familiar rather than unfamiliar. In other words, familiar faces appeared to be less susceptible to serial dependence than were unfamiliar faces. Experiment 2 yielded the familiar face immunity across all participants regardless of recognition sensitivity, while in Experiment 1 familiarity only affected serial dependence when participants were better able to distinguish the learned faces.

### 6.1. Serial dependence and familiarity

Both experiments demonstrated that familiar faces could be less susceptible than unfamiliar faces to the inter-trial attractiveness effect, taken here to be an indicator of serial dependence. Experiment 2 found no evidence that there was any perceptual pull from the previous face on the percept of familiar faces, as faces rated after attractive faces did not significantly differ from those rated after unattractive faces. This is strong evidence in favour of the hypothesis that familiarity with faces modulates the effects of serial dependence. To the best of our knowledge, this finding is the first evidence that face perception may exploit familiarity in this way. This extends the body of the literature which demonstrates that familiar face identification is robust over different contexts and further confirms that familiar faces are processed in a qualitatively different manner from that for unfamiliar faces (e.g. [2]).

How does the visual system profit from being able to accurately perceive identity despite image variation? Familiar faces generally belong to people that we frequently encounter and who therefore bear some significance to us. As a result, seeing a familiar face would be a reliable signal that a meaningful interaction is about to occur. Thus, it would be adaptive for the visual system not to average the current face with a previous signal from a different identity. The increased resilience of familiar faces against temporal integration could further allow the visual system to prioritize the processing of a familiar face, and accordingly drive spatial attention and resources towards processing that face. Familiarity may equally mean that such faces can be processed more easily—that is, with less neural effort as suggested by Rossion *et al.* [44,50]—given the extent to which we have encountered them before. By contrast, the visual system appears to be more likely to compress and ultimately confuse briefly seen unfamiliar faces with other faces. This corresponds with the well-documented finding that we distinguish between unfamiliar faces poorly [20,51,52].

The mechanism by which the visual system achieves this stable percept of familiar faces remains to be confirmed. However, the present findings are consistent with the theorized existence of stored visual representations for each familiar face (e.g. [53–55]). Indeed, the fact that this immunity could be induced by training participants with repeated exposure to a particular face (in Experiment 1) implies that our visual representations of familiar faces are enough to override the effect of serial dependence and anchor the way in which we process faces. While participants may have been primed with additional semantic and affective information when viewing famous faces, the only experience they had with the faces in Experiment 1 before the adaptation stage was the learning and recognition tasks over the course of an hour, with no other semantic information provided. This limited exposure probably explains why familiarity only affected serial dependence for participants who were better able to distinguish the newly learned faces in Experiment 1.

### 6.2. Serial dependence in attractiveness ratings

The robust serial dependence observed for unfamiliar faces in both experiments converges with the results of Kondo *et al.* [36,37], Kramer *et al.* [38] and Taubert *et al.* [39]. In the recent literature, however, there has been criticism as to whether this sequential effect is actually indicative of a perceptual phenomenon. Instead, past studies have largely attributed these effects to response biases resulting from repetitive key-presses (motor response bias) or a pattern of responding dependent on the last judgement made regardless of the actual stimuli presented (cognitive decisional strategies). Though the involvement of such biases can never be absolutely eliminated from any such experiment, several methodological considerations and significant analytical observations mean that the present results cannot be accounted for solely on this basis.

The present research made several methodological improvements on past investigations which sought to decrease the development and influence of any motor biases. In particular, participants rated attractiveness by adjusting a bar on a continuous scale devoid of numerical anchors instead of pressing keys corresponding to discrete Likert scales, as used in many previous investigations [36–38,40]. More recently, Taubert *et al.* [39] used a binary decision task to mimic an Internet dating task, but this limited the impact of serial dependence because any given stimulus could not be ‘more attractive’ than a previous positive response (or *vice versa* for unattractive faces). In addition to this loss in sensitivity, a binary decision is more vulnerable to repetitive response patterns (i.e. participants simply responding with the same button over and over). The sliding bar approach we used here had a cursor that appeared in a random starting position upon the initiation of each trial, making it difficult to respond repetitively in a way that would result in a systematic pattern consistent with the *n *− 1 effect. If the participant, for example, held the arrow key for the same amount of time, it would only serve to increase noise in the data. Using short blocks (40 trials per block) and breaks between each block aimed to minimize the development of cognitive strategies. Future research may benefit from considering alternative statistical analysis methods such as regression of attractiveness scores on previous trials to further consider variance in attractiveness scores.

Most importantly, familiarity with the current face alone appeared to modulate the inter-trial attractiveness effect. In both experiments, there was a systematic reduction in the *n *− 1 effect when the current face was familiar, with no evidence in Experiment 2 of any assimilation in attractiveness ratings when the current face was familiar. As the *n* − 1 effect was reduced only in the condition with sound theoretical reasons predicting this diminished effect, it is highly unlikely to have been driven by a systematic response pattern as this would be expected to produce the *n* − 1 effect across all conditions. To explain our result in terms of cognitive strategy is untenable as it would involve a constantly switching decisional approach. This seems highly unlikely given that the face order was unpredictable—familiar faces were randomly interleaved with novel faces—and an account in terms of systematic response bias would require observers to maintain separate streams of previous faces and continuously switch between different strategies based on familiarity. There could be a concern that participants were actively remembering their previous ratings of familiar faces and responding consistently. While this cannot be completely ruled out of any experiment of this nature, this possibility was reduced by having a continuous response scale (with initial cursor position selected at random) because it would have been difficult for participants to consciously recreate their previous response. Thus, the observation that familiarity with the target face affected serial dependence indicates that participants were reacting to the stimulus when making attractiveness ratings rather than relying on a cognitive decisional bias unrelated to the stimulus.

Overall, the observed inter-trial effects provide persuasive empirical evidence that the perception of attractiveness of unfamiliar faces is at least partially stabilized by a mechanism that pools samples over time. Past perceptual experience has frequently been found to confer an advantage in the present, and often does so by influencing our current experience of the world (e.g. recall from visual memory: [56]). For instance, several experiments conducted by Maljkovic & Nakayama [57–59] demonstrated ‘priming of pop-out’ effects where responding to a certain stimulus feature (e.g. shape) was facilitated by the repetition of a task-irrelevant pop-out feature (e.g. colour). Hysteresis is another perceptual phenomenon in which our visual perceptual system resolves situations of suboptimal or ambiguous visual conditions by biasing current stimuli towards the appearance of previous stimuli [60]. Generally, our perceptual history is thought to influence current experience because perception is an active and predictive phenomenon, and recent experience in particular is a reasonable predictor of the present state of the world [61–63].

The present results are consistent with the occurrence of serial dependence in the perception of facial identity [33]. The observed serial dependence in attractiveness ratings provides evidence that serial dependence operates to stabilize perception of important characteristics associated with facial identity. The visual system is constantly overwhelmed with information and variation while facial identity remains static over the course of a normal encounter, making it adaptive in most circumstances to stabilize identity, along with other typically stable attributes such as gender and attractiveness, by combining the current with the previously seen face. Thus, it is unsurprising that serial dependence generalizes to attractiveness, a central social characteristic that is unlikely to change dramatically during a single encounter after an initial appraisal. It would be maladaptive for the visual system to repeatedly sample the face attractiveness when it is a fundamentally stable attribute. Rather, by assuming continuity after an initial appraisal, the perceptual system profits from a better estimate of attractiveness by pooling samples from the recent past.

The finding that perceived attractiveness can be influenced by familiarity opens up many interesting future avenues of research into the interaction between familiarity and serial dependence. For instance, it may be that familiar faces are completely excluded from serial dependence computations, in which case there would also be reduced serial dependence in faces which follow familiar faces. Furthermore, research could also manipulate the familiar faces presented to participants, e.g. by changing facial viewpoint, lighting or other external condition changes, to discern any effect on the presence or absence of any serial dependence effect in familiar faces.

In sum, the current research contributes to our understanding of both serial dependence and the effects of familiarity on face perception. The results indicate that the perceived attractiveness of a face is influenced at least in part by the attractiveness of a previously seen face. Furthermore, they present the first evidence that the recognition of familiar faces can be resistant to the temporal averaging revealed by positive serial dependencies.

## Supplementary Material

Kok2015_Experiment1LEARN_raw

## Supplementary Material

Kok2015_Experiment2CELEB_raw

## Data accessibility

The raw data have been made available as the electronic supplementary material.
